# Translation and validation of the Persian version of Godin Leisure-Time Exercise Questionnaire in patients with multiple sclerosis

**DOI:** 10.1186/s12883-021-02465-5

**Published:** 2021-11-05

**Authors:** Mohammad-Reza Fattahi, Pardis Noormohammadpour, Meysam Ramezani, Mohammad Ali Sahraian, Mohammad Ali Mansournia, Mastaneh Rajabian Tabesh, Mohamed Ali Mesgarof, Maryam Abolhasani

**Affiliations:** 1 Multiple Sclerosis Research Center, Neuroscience institute, Tehran University of Medical Sciences, Tehran, Iran; 2 Sports Medicine Research Center, Neuroscience Institute, Tehran University of Medical Sciences, Tehran, Iran; 3 Department of Sports and Exercise Medicine, School of Medicine, Tehran University of Medical Sciences, Tehran, Iran; 4 Sports and Exercise Medicine, MS Research Center, Neuroscience Institute, Tehran University of Medical Sciences, Tehran, Iran; 5 Neurology, MS Fellowship, MS Research Center, Neuroscience Institute, Tehran University of Medical Sciences, Tehran, Iran; 6 Department of Epidemiology and Biostatistics, School of Public Health, Tehran University of Medical Sciences, Tehran, Iran; 7 PHD Candidate in Sport Physiology Cardiac Primary Prevention Research Center (CPPRC), Heart Centre hospital, Tehran University of Medical Sciences, Tehran, Iran; 8 Department of Sports Medicine, Sports and Exercise medicine, Sina MS Research Center, Sina Hospital, Hassan Abad Square, Tehran, Iran

**Keywords:** Multiple sclerosis, Cross-cultural comparison, Persian, Validation study, Reliability and validity

## Abstract

**Study Design:**

Psychometric study.

**Objective:**

The purpose of this study is to translate, culturally adapt and evaluate the validity and reliability of the Persian (Farsi) version of GLTEQ in patients with multiple sclerosis.

**Methods:**

This study had three phases, including translation of the questionnaire into Persian and making cultural adaptation, evaluation of pre-final version of questionnaire’s comprehensibility in a pilot study, and investigation of reliability and validity of the final version of the translated questionnaire. Content validity, and convergent validity (correlations among the Persian version of GLTEQ and Global physical activity questionnaire (GPAQ), and international physical activity questionnaire (IPAQ)) and after all test-retest reliability were studied.

**Results:**

The subjects were 87 MS patients. The Persian version demonstrated moderate to good convergent validity; the correlation coefficient between the Persian version and GPAQ was r=0.64 (p<0.001), and between the Persian version and IPAQ was r=0.59 (p<0.001). The test-retest reliability was strong (Intra-class Correlation (ICC) value ranged between 0.908 and 0.992). Besides, its face validity and content validity were acceptable.

**Conclusions:**

The Persian version of GLTEQ is a valid and reliable instrument to assess physical activity in patients with MS. This questionnaire can be a step toward standardization of physical activity measurement in patients with MS. Also, in research, it provides the possibilities to carry on a comparative study across cultures using the same outcome measure.

**Supplementary Information:**

The online version contains supplementary material available at 10.1186/s12883-021-02465-5.

## Introduction

Pathological changes in multiple sclerosis (MS) cause many complications and problems such as sensory, motor, visual, and autonomic symptoms for patients [1, 2]. Although the onset of the disease is transient, and the patient’s problems improve with re-myelination over time, myelin regeneration becomes less and less with the onset of pathological changes. Finally, disability could affect different aspects of their life. Thus, progression of disability could decrease the level of physical activity and its intensity. Therefore, assessment of physical activity could indirectly estimate the level of disability [3–6].

MS is one of the most common neurological diseases in some countries like Iran [2, 7]. In 2008, 2.1 million people worldwide were involved [1, 8, 9].The prevalence of MS is dramatically rising in Iran [10, 11], so Sahraian et al. declared that in Tehran, it was estimated at 52 per 100,000, of which 72.3 % were female, and 27.7 % were male [12].

It has been a long time that MS patients are considered less active than healthy matched subjects in the community [13–15]. Adverse effects of inactivity, such as the increased risk of osteoporosis (decreased bone mineral density), depression and death caused by cardiovascular disease, have been reported in MS patients. As the disease progresses, symptoms such as dysfunction, disability, and decreased quality of life eventually worsen. On the other hand, disease-related symptoms associated with locomotor system such as weakness of limbs, muscle spasms, and balance problems could escalate patients’ inactivity. Fatigue is the biggest challenge for everyday tasks, which creates many limitations [16–18].

Despite the recommendation of medical professionals, MS patients do not have the proper level of physical activity, necessary for their condition improvement [19, 20]. Even more, MS patient’s exercise goals should be individually set based on patient’s characteristics and physician consultations considering the results of preliminary exercise tests [18].It is challenging to apply objective methods like pedometers and accelerometers to measure physical activity level [21]; In this case, self-report measurements such as valid and reliable questionnaires could be a contributor [22, 23].

Godin Leisure-Time Exercise Questionnaire is a self-administered questionnaire, and its administration requires the questionnaire to be translated into the language of population under study, Persian (Farsi) in this case. Also, as the Iranian culture is different from the questionnaire’s country of origin, it seems that cultural adaptation and validation should be considered. Thus, after considering these points, the results of the study with such a questionnaire could be comparable and applicable internationally.

This study investigated the validity and reliability of the Persian version of GLTEQ in MS patients and make it credible among Persian speaking areas.

## Materials and methods

This study was a cross-sectional study conducted to translate, culturally adapt and evaluate the validity and reliability of the Persian (Farsi) version of GLTEQ in patients with MS.

The inclusion criteria include being over the age of 18, diagnosed with MS for more than a month, and being able to read in Persian. The subjects should not have a history of attack or relapse or being treated with corticosteroids or psychological drugs in the past month. They should be able to walk at least 100 steps without walking aids.

They should not have orthopedic, rheumatic or neurological or psychological disorders that affect physical activity and/or participated in a rehabilitation program in the past month.

The exclusion criteria were obstacles to practice aerobic activities such as orthopedic and muscular problems, being a smoker, pregnancy, participating in a rehabilitation program, lack of access to the study site due to the disability of patients, illiteracy and reluctance to fill out the questionnaire.

Estimated sample size based on the convergent validity and the following values are calculated to be 85 subjects according to the following formula [24].

$$\frac{{\left(\left({Z}_{1-\frac{\alpha }{2}}\right)+\left({Z}_{1-\beta }\right)\right)}^{2}}{{\left(\frac{1}{2}\text{ln}\left(\frac{1+{\uprho }}{1-{\uprho }}\right)\right)}^{2}}+3=85$$

(z_1_= Critical Value; α = Significance =0.05; β = Confidence =0.2; ρ = power=0.3)

### Study protocol

This study was conducted in three general stages:

First, the original questionnaire in English was translated into Persian, and necessary cultural adaptations were applied. In the next step, during a pilot study, the level of understanding of the original version of the Persian questionnaire was examined. After all, validity and reliability of the latest version of the questionnaire were evaluated. The study protocol was approved by Tehran University of Medical Sciences ethics committee (ethic code: IR.TUMS.MEDICINE.REC.1397.966). The study aims and steps were explained for the participants and they were recruited into the study after signing the written informed consent forms. The study was conducted in accordance with the standards of 1964 Helsinki Declaration and its later amendments.

### Study procedures and outcome measures

MS patients who met the inclusion criteria were recruited into the study. This study was conducted in the MS Research Center of Sina Hospital, between September 2018 and October 2019, and 87 subjects entered the study following a neurologist approval.

The demographic data of the participants were obtained and their weight and height were measured. Then, they completed the Persian version of GLTEQ. Comparator scales described below were completed based on their instructions.

To assess GLTEQ, the authors proposed the following research questions(R) and hypothesis(H): first, the translated GLTEQ items are clear and easy to understand for patients (R1); second, the items of GLTEQ are relevant and appropriate in terms of assessment of exercise based on the expert panel opinion (R2); third, there are moderate to strong correlations between item answers after two weeks (H1); fourth, results of GLTEQ have a moderate to good correlation with other questionnaires that asses the level of exercise and physical activity like GPAQ and IPAQ (H2).

### Comparator scales

#### Godin Leisure-Time Exercise Questionnaire (GLTEQ)

This questionnaire has two questions, three sections of the first question report “how many times on the average does the patient do the strenuous, moderate, and mild/light forms of exercise for more than 15 minutes during their free time in a typical week (7-Day period)”.

The second question asks, “how often does the patient engages in a regular activity long enough leads to sweat (rapid heart bits) during a typical week (7-Day period).”

The GLTEQ score summarizes the points given to strenuous, moderate, and mild/light physical activities, is 9, 5, and 3 respectively. The overall score is between zero and 119, and higher GLTEQ scores demonstrate more physical activity.

#### Persian version of Global Physical Activity Questionnaire (GPAQ)

The GPAQ questionnaire consists of 16 questions and collects information on the frequency and time of physical activity in three sections including work, relocation, and recreational activities, plus sedentary behaviors in a week. The results are based on METs (Metabolic Equivalent of Tasks) as an objective measure of expending energy, relative to the weight of a person which divides people in three groups of low, moderate, and high physical activity level [3, 25]. In many national multi-central surveys in physical activity, the Persian version of this questionnaire which is both valid and reliable is being used [26–28].

#### International Physical Activity Questionnaire (IPAQ)

The IPAQ consists of 27 questions and examines physical activity in the following areas during a week: leisure physical activity, home and yard activities (gardening), work-related physical activity; physical activity related to transferring, and questions about the details of types [29]. The results are based on MET–minutes per week and higher scores mean a higher level of physical activity level. The Persian version of this questionnaire is valid and reliable [30].

### Psychometric properties assessment

#### Face validity assessment

Patients in the pilot study group were asked to express their understanding regarding each question after they completed the GLTEQ. After the patients expressed their perceptions of the meaning of each question, the researcher checked their understanding of the original aim of the question and if they were a match, the question was considered to be understood correctly by the patient (answer to R1). Then, the percentage of participants whose perceptions of a particular question contradicted the real purpose of the question was calculated and recorded as a misunderstanding index [31, 32]. If any item has a misunderstanding index above 20 %, changing questionnaire wording would be necessary [32].

$$Misunderstanding index=\frac{Number of participants whose perceptions of a particular question contradicted the real purpose of the question \times 100}{Number of all participants}$$

#### Content validity assessment

To assess the content validity, members of the expert panel were asked to rate each question based on four Likert scales including 1: not relevant, 2: unable to assess relevance without item revision, 3: relevant but needs minor alteration, 4: very relevant). Then, CVI (Content Validity Index) was calculated for each question based on the fraction of experts who select ratings 3 and 4 to all (answer to R2) [33].

$$CVI=\frac{Number of experts selecting ratings 3 and 4 }{Number of experts}$$

#### Convergent validity assessment

Convergent validity investigates whether the questionnaire assesses variables it should assess. Thus, correlations among the Persian version of GLTEQ, Global physical activity questionnaire (GPAQ), and international physical activity questionnaire (IPAQ)) were studied (check H2).

#### Reliability assessment

To determine reliability (test-retest), 50 randomly selected patients out of 87 completed the GLTEQ after two weeks. Patients’ clinical status was checked after two weeks and considered before reassessing reliability. The intra-class correlation coefficient based on the one-way random-effects model for each question of GLTEQ was conducted to assess the test-retest reliability (check H1).

Godin Leisure-Time Exercise Questionnaire has two questions. The first question asks about participation in exercises, and question two asks about participation in regular activities. As it seems, these two questions cover different topics, and they are inconsistent in essence.

Also, question one has three parts asking about different exercise intensities, including strenuous, moderate, and mild exercise. As it is evident, these three parts are also inconsistent intrinsically.

Therefore, the authors did not measure Cronbach’s alpha for the whole questionnaire, but ICC was reported for each question.

#### Translation and Cultural adaptation

Based on the existing guidelines on translation and matching [34, 35], two native Persian translators translated the questionnaire from English to Persian, independently; they were asked to translate with more emphasis on keeping the content of the questions, rather than conducting a literal translation. The first translator was a physician who knew the purpose of the study and translation, and the other was an official translator who had no idea of the study and its purpose. After completing the translators’ work, the two versions of translation were compared, and the two translators examined their contradictions and differences; then, a single translation of the questionnaire was obtained. In the next step, two native Persian-speaking translators with history of living in English-speaking countries for a long time, translated the Persian translation into English without knowing the purpose of the study.

Afterwards, in an expert committee consisting of various members, including an epidemiologist, two neurologists (one has a fellowship in MS), a general practitioner, two sports medicine specialists and two academic translators, the differences between English translations and the main questionnaire were reviewed and discussed. At the end, none of the items has been changed in Persian version of the questionnaire.

### Pilot Study

In the next step, the semi-final Persian questionnaire was given to 50 MS patients to examine the comprehensibility of the questions, and find out whether the questions measure the same concept they planned to measure.

In this process, the patients were asked to complete the questionnaire which took a maximum of 10 min; then, they were asked about their interpretation of each question and the answer given to that were recorded. They were asked to identify words or phrases that were difficult for them to understand, and to express their general opinion on the questionnaire. If there was a difference between the patients’ concepts and what the questions were planned to measure, this number was used to calculate the misunderstanding index. Moreover, the expert committee supervised the concise translation considering the cultural adaption of GLTEQ. Finally, after everyone’s agreement and based on its content and face validity, the final version of the Persian questionnaire (Additional file 1) was approved  by the Committee of Experts.

#### Statistical Analysis

All analyses were performed via SPSS 21 software (SPSS Inc, Chicago, IL). The quantitative data were presented as mean (SD) and median (IQR) and categorical variables were described as number (percent). The intra-class correlation coefficient based on the one-way random-effects model for each question of GLTEQ was conducted to access the test-retest reliability, Misunderstanding Index, and CVI, measured face and content validity, respectively. To assess the convergent validity Correlations among the Persian GLTEQ score and Persian versions of GPAQ and IPAQ (which are valid and reliable) we used the Spearman’s rank correlation coefficient to test convergent validity.

## Results

### Demographic and baseline data

Five patients were excluded from the study if they were reluctant to complete the questionnaire after two weeks, or due to causes like experiencing an attack, initiation of a new exercise, participating in a rehabilitation program, musculoskeletal injury or acute illness. Finally, out of 92 patients, 87 were completely enrolled. Their demographic information and symptom characteristics are presented in Tables 1 and 2.

The range of Expanded Disability Status Scale (EDSS) of our participants was 2 to 5.5.

**Table 1:** Socio-demographic characteristics of the study population

*Quantitative variables*	*Mean*	*SD*	*Range*
*Age (years)*	*37.5*	*8.8*	*15-60*
*Weight (kg)*	*71.1*	*11.0*	*49-92*
*Height (cm)*	*165.3*	*8.4*	*150-186*
*BMI*^a^* (kg/m2)*	*26.0*	*3.0*	*19.1-32.4*
*Gender*	*Frequency (n)*	*Percentage (%)*	
*Male*	*30*	*33.7*	
*Female*	*57*	*64*	
*Level of education*			
*High school*	*4*	*4.5*	
*Diploma*	*16*	*18*	
*Pre-university*	*7*	*8*	
*Bachelor’s Degree*	*44*	*49.4*	
*Master’s Degree*	*7*	*8*	
*Professional PhD*^b^	*3*	*3.4*	
*Doctorate*	*6*	*6.7*	

^a^BMI: Body Mass Index, ^b^ Professional PhD: (Medicine, Dentistry, Veterinary Medicine, Law), SD: Standard deviation

**Table 2:** Characteristics of MS symptoms

		*Frequency (n)*	*Percentage (%)*
*Type of MS*	*Relapsing remitting*	*41*	*46.1*
	*Secondary progressive*	*46*	*51.7*
*Disease duration(years)*	*< 1*	*0*	*0*
	*1-2*	*5*	*5.6*
	*2-3*	*6*	*6.7*
	*>3*	*76*	*85.4*

Since the questions ask about the frequency of participation in exercise and provide the name of different sports familiar for the patients in the pilot study, the researchers did not change any item. The results of GLTEQ in our participants are presented in Table 3. Based on GLTEQ, most of our participants were sometimes active (64 %), and the mean (SD) of Godin’s score was 29.7 ± 15.7.

**Table 3:** Godin leisure exercise questionnaire results in the study population

*Weekly exercise*	Median (Interquartile range)
strenuous *activity*	0 (0, 0)
*moderate activity*	2 (0, 3)
*mild activity*	5 (3, 7)
*Normal weekly activity*	Frequency (%)
*mostly*	17 (19.1)
*sometimes*	57 (64)
*Never*	13 (14.6)
*Godin’s total score*	Mean (SD) 29.7 (15.7)

Godin's score:  summation of intense, gentle and light activity values with the weights of 9, 5 and 3 respectively

SD: standard deviation

### Test-retest

#### Reliability

Test-retest reliability is calculated separately for each question, and the results are presented in Table 4. Based on these findings, all questions in the Persian version of *GLTEQ* have excellent reliability.

Godin Leisure-Time Exercise Questionnaire has two questions. Question one asks about participation in exercises, and question two asks about participation in regular activities. As it seems, these two questions ask about different subjects, and they are inconsistent, in essence. Also, question one has three parts about different exercise intensities, including strenuous, moderate, and mild. As it is evident, these three parts of question one are also inconsistent intrinsically.

Therefore, the authors did not measure Cronbach’s alpha for the whole questionnaire, but ICC was reported for each question.

It is worth noting that intra-class correlation coefficient (ICC) represents the amount of reliability; values are divided into the following groups: less than 0.5, between 0.5 and 0.75, between 0.75 and 0.9, and greater than 0.90, defined as poor, moderate, good and excellent reliability, respectively [36]. (Table 4)

**Table 4:** ICC of the 4 questions of the Persian version of GLTEQ in two weeks

*Questions*	*ICC (r)*	*95 % CI*
*Q1A (Intense exercise)*	*0.992*	*0.985-0.995*
*Q1B (Gentle sports activities)*	*0.992*	*0.985-0.995*
*Q1C (Light sports activity)*	*0.926*	*0.875-0.957*
*Q2 (Ordinary weekly activity)*	*0.908*	*0.846-0.947*
*Total GLTEQ score*	*0.981*	*0.966-0.989*

ICC: Intraclass Correlation Coefficient; CI: confidence interval

#### Face Validity

Using the misunderstanding index, the face validity of the Persian version was examined after the pilot study; the misunderstanding index of every item was<20 %. Also, the average time spent on answering the questionnaire was about 5 min.

#### Content Validity

To evaluate the content validity, CVI was calculated for each question, and it was 1.0 for all questions. Based on the previous study[37], the CVI for all GLTEQ questions was above the essential value[33].

#### Convergent Validity

In this study, spearman’s rank correlation coefficient between 0.1 and 0.3, 0.4 to 0.6, 0.7 to 0.9, and 1.0 were considered weak, moderate, strong, and perfect, respectively[38].

There was a moderate and strong positive relationship between the Persian version of GLTEQ and GPAQ, IPAQ[39]. (Table 5)

**Table 5:** Spearman’s rank correlation coefficient of Persian version of GLTEQ with GPAQ, IPAQ

Questionnaire	Correlation	P-value after Bonferroni correction
GPAQ	0.64	<0.001
IPAQ	0.59	<0.001

GPAQ: Global Physical Activity Questionnaire; IPAQ: International Physical Activity Questionnaire; GLTEQ: Godin leisure Time Exercise questionnaire

The parameters and structure of our questionnaire were equivalent to its English counterpart.

## Discussion

It has been proven that exercise in MS patients has favorable outcomes in their quality of life [40, 41]. Thus, recently, clinicians and researchers have focused on promoting physical activity in MS patients enthusiastically [42, 43]. They reached the fact that these patients receive more benefits in cardiorespiratory, endurance, muscle strength, bone health, flexibility, balance, and tiredness [20, 44]. Notably, they have seen that in adults with neurological disorders, physical activity contributes to the elimination of depressive symptoms[45]. Currently, GLTEQ is the most practical and widely used tool for evaluating physical activities in epidemiological research in patients with MS[46, 47]. It also has been qualified for pediatric-onset MS by using the accelerometer (as an objective index). Moreover, its validity has been shown in vigorous and moderate-to-vigorous physical activity levels [48]. Subsequently, GLTEQ can facilitate the understanding of patient’s physical activity level profile in order to enable the possibility of improvement in MS patient’s outcomes. This study contributes to the evaluation of translation, reliability and validity of the Persian version of GLTEQ in patients with MS for the first time in Iran.

There are studies that compare this questionnaire with other tools such as Stanford Leisure-Time Activity Categorical Item and American College of Sports Medicine (ACSM) fitness guidelines [49, 50]. But, to apply a research questionnaire in different languages or cultures, translation into the target language must be applied. Also, cultural adaptation depends on the questionnaire’s questions and the result of the study to maintain the content validity[51]. This questionnaire has been validated for different age-group patients [52] as well as other languages[52, 52].Furthermore, this study’s Test-retest results concur the fact that reliability of the Persian version of GLTEQ is excellently comparable to the English version[3]. Moreover, this study shows the correlation between different scores (ICC=0.981) which demonstrates excellent reliability and content validity index (1.0). Similarly, these results have been obtained in Turkish Godin Leisure-Time Exercise Questionnaire (0.98) in 2016 with ICC and CVI of 0.98 and 0.82, respectively[53].

On the other hand, a narrative review published by E.M. Sikes et al. in 2019, concluded that GLTEQ is a valid self-report measure of physical activity in patients with MS as a reliable measurement of physical activity level.

In the future, this questionnaire could be used as an appropriate, simple, and effective tool to determine changes in outcome and pattern of physical activities, limited to a post effective intervention[43]. Also, in 2018, Motl et al. explained that th GLTEQ primarily operates based on moderate to vigorous physical activity rather than sedentary lifestyle in patients with MS and is a valid tool for assessing physical activity compared to the use of accelerometer among the said population[55]. In another study, Katrina D. DuBose et al. aimed to validate the modified version of the Godin-Shephard Leisure-Time Exercise Questionnaire. They indicated that moderate-to-vigorous activity results can be deceptive because of invalid results in population groups of middle-aged patients [55]. In conclusion, even though GLTEQ could have some limitation in certain groups of patients [53, 55], the Persian version presented in this study can be carried out for Persian speaking MS patients. Still, GLTEQ is the simple and effective enough to measure the activity level of the patients effectively [53, 55].

After all, this questionnaire would provide clinicians and researchers with a valid measurement tool for physical activity research in MS patients who speak Persian.

### Limitations

A number of cases were excluded during the study due to their uncontrollable fatigue, low literacy, MS attack, or evolution in the course of treatment. Besides, some did not have access to the research site due to their disabilities.

Patients faced recall bias during the process of completing the questionnaire. Also, data-gathering in the tertiary medical center made the study prone to selection bias.

Furthermore, inconsistency between questions one and two in this questionnaire did not let the authors measure Cronbach’s alpha for the whole questionnaire.

The authors were unable to perform the known-groups validity assessment considering the fact that discriminative variables were absent form questionnaire’s assessment construction.

## Conclusions

The Persian version of GLTEQ is a valid and reliable tool to assess leisure-time physical activity in patients with MS. This Persian version of the data-matching questionnaire could be a useful step in standardizing studies on physical activity in MS patients and, consequently, helps us to effectively determine the level and pattern of physical activity, and perform sports intervention among these patients.

This study could also aid us in creating new research avenues, such as comparative studies among Persian speaking researchers using this study’s version of GLTEQ.

## Supplementary Information

**Additional file 1.**

## Data Availability

The datasets used and/or analyzed during the current study are available from the corresponding author on reasonable request.
